# Invariant natural killer T cells in lung diseases

**DOI:** 10.1038/s12276-023-01024-x

**Published:** 2023-09-11

**Authors:** Dongjin Jeong, Yeon Duk Woo, Doo Hyun Chung

**Affiliations:** 1 Laboratory of Immune Regulation in Department of Biomedical Sciences, Seoul National University College of Medicine, Seoul, Korea; 2 Department of Pathology, Seoul National University College of Medicine, Seoul, Korea

**Keywords:** NKT cells, Mucosal immunology

## Abstract

Invariant natural killer T (*i*NKT) cells are a subset of T cells that are characterized by a restricted T-cell receptor (TCR) repertoire and a unique ability to recognize glycolipid antigens. These cells are found in all tissues, and evidence to date suggests that they play many immunological roles in both homeostasis and inflammatory conditions. The latter include lung inflammatory diseases such as asthma and infections: the roles of lung-resident *i*NKT cells in these diseases have been extensively researched. Here, we provide insights into the biology of *i*NKT cells in health and disease, with a particular focus on the role of pulmonary *i*NKT cells in airway inflammation and other lung diseases.

## Immunology: cellular gatekeepers of pulmonary inflammation

A small subset of immune cells residing in the lung plays a pivotal part in governing the inflammatory response in a range of pulmonary disorders. Invariant natural killer T (*i*NKT) cells monitor the blood vessels in the lung for infection or other sources of tissue damage, which cause the cells to release chemical signals that rally a broader immune response. Doo Hyun Chung of Seoul National University, South Korea, and colleagues review the complex role of *i*NKT cells in determining the severity and outcome of respiratory disorders ranging from flu to pulmonary fibrosis. Sometimes *i*NKT cells are protective, for example by helping to quell the damaging inflammatory response associated with pulmonary fibrosis. However, some studies suggest that *i*NKT cells can exacerbate certain forms of asthma, highlighting the complexity of their physiological function.

## Introduction

NKT cells are a unique subset of T cells that share features of both conventional T cells and natural killer cells. Unlike conventional T cells, NKT cells express TCRs that recognize glycolipid antigens loaded on CD1d, which is a nonpolymorphic major histocompatibility complex (MHC) class I-like protein^1^. NKT cells are classified into two distinct subsets (types I and II) on the basis of their TCR repertoire. Type I NKT cells express semi-invariant TCRβ chains combined with an invariant TCRα chain (Vα14 chain in mice and Vα24 chain in humans); these cells are, therefore, also called *i*NKT cells. They respond particularly strongly to α-galactosyl ceramide (α-GalCer), a marine sponge-derived glycolipid. In contrast, type II NKT cells have diverse polyclonal TCR repertoires that recognize lipid antigens such as sulfatide^2^. Studies on type II NKT cells are very limited due to the rarity of these cells and their lack of clear surface markers^3^. In contrast, *i*NKT cells have been extensively studied in many contexts because they are relatively abundant and can be readily identified with α-GalCer-loaded CD1d tetramers. The remainder of this review will focus on *i*NKT cells.

*i*NKT cells are largely sessile cells that reside in most tissues, including nonlymphoid tissues such as the liver, lungs, intestine, urogenital tract, adipose tissue, and skin^4^. However, their frequency relative to other lymphocytes varies depending on the tissue^5,6^. Thus, in mice, the lymphocytes in the liver are dominated by *i*NKT cells (10–30% of all lymphocytes). *i*NKT-cell dominance is also observed in murine adipose tissue (2–8% of all lymphocytes, 8–12% of adipose T cells), lungs (5–10% of all lymphocytes), and spleen (1–3% of all lymphocytes)^6,7^. In humans, *i*NKT cells are much less frequent in the liver (0.05–1% of all lymphocytes)^8^ and spleen (0.5–2% of all lymphocytes). However, they represent 10–25% of the T cells in adipose tissue, similar to their frequency in murine fat^9^. In contrast, the blood and thymus of both mice and humans contain low frequencies of *i*NKT cells (0.5–2%)^10^.

Although *i*NKT cells account for only ~1% percent of circulating T cells overall^10^, they often have a powerful immunological effect because of their abundant secretion of cytokines a few hours after activation of their TCR. Their cytokines include T-helper type-1 (Th1) cytokines, namely, interferon-gamma (IFN-γ) and tumor-necrosis factor-α (TNF-α); T-helper type-2 (Th2) cytokines, namely, interleukin (IL)-4 and IL-13; T-helper type-17 (Th17) cytokines, namely, IL-17A and IL-22; and the regulatory cytokine IL-10^11^. These cytokines impact various immune cells, ranging from innate immune cells such as macrophages, dendritic cells (DCs), and natural killer cells to adaptive immune cells such as T and B cells. Thus, *i*NKT cells can regulate both innate and adaptive immune responses. These regulatory activities have been found to play a role in many diseases, including rheumatoid arthritis^12^, asthma^13^, tumors^14^, and infectious diseases^15^.

## Thymic development of *i*NKT cells

Similar to conventional T cells, *i*NKT cells develop in the thymus via four double-negative stages^16^ that eventually lead to TCRα chain-expressing CD4^+^CD8^+^ double-positive (DP) thymocytes. These cells then undergo positive selection with CD1d-presented glycolipids on DP thymocytes^17^. This differs from the development of conventional T cells, which are instead positively selected by MHC-presented peptides on thymic epithelial cells. *i*NKT-cell positive selection is driven by not only TCR activation but also costimulation signals from the signaling lymphocytic-activation molecule (SLAM) receptor on nascent *i*NKT cells; these signals induce the expression of the transcription factor early growth response protein 2 (Egr2), which is dispensable for conventional T-cell development^18^. Positively selected *i*NKT cells then enter unique development stages. The earliest is stage 0, which is characterized by CD24 expression. Stage 0 cells transition into the next stages, which can be defined differently depending on the proposed model. The linear maturation model, which is based on the expression of surface molecules such as CD24, CD44, and NK1.1, proposes that stages 1, 2, and 3 are characterized by CD24^lo^CD44^hi^NK1.1^−^, CD24^lo^CD44^lo^NK1.1^−^, and CD24^lo^CD44^hi^NK1.1^+^ phenotypes, respectively. In contrast, the lineage differentiation model, which is defined by transcription factor expression and cytokine production, proposes that stage 0 cells (NKT0s) develop into NKT1, NKT2, NKT17, and NKT10 subsets^19–22^. Thus, T-bet^+^ *i*NKT1s secrete Th1 cytokines (IFN-γ and TNF-α), Gata3^+^ *i*NKT2s produce Th2 cytokines (IL-4 and IL-13), and RORγt^+^ *i*NKT17s generate Th17 cytokines (IL-17A and IL-22)^23–25^. With regard to *i*NKT10s, their signature transcription factor remains unknown, but they secrete the anti-inflammatory cytokine IL-10^21,26,27^.

Recently, several groups employing single-cell RNA sequencing and unbiased computational analysis have proposed a third model that is based on transcriptional profiles and integrates both of the previous models. Thus, *i*NKT0 cells express *Sox4, Lef1*, and *Id3*, and their surface molecule phenotype is similar to that of stage 0 and 1 *i*NKT cells, namely, CD24^+^CD44^–/lo^NK1.1^−^. *i*NKT1 cells express *Ifng*, *Tbx21*, *Xcl1* and *Il2rb* and bear the stage 3 surface phenotype (CD24^lo^CD44^hi^NK1.1^+^). *i*NKT2 cells express *Il4*, *Gata3*, *Icos*, and *Zbtb16*, and their surface molecule phenotype is that of stage 2 cells (CD24^lo^CD44^lo^NK1.1^−^). *i*NKT17 cells express *Il17a*, *Rorc*, *Ccr6*, and *Itgb7* and bear the stage 2 surface phenotype^23,24,28–30^. Several studies have also shown that the thymic development of *i*NKT cells, but not conventional T cells, is regulated by specific cytokines (IL-15 and GM-CSF), SAP-Fyn signaling, other transcription factors (PLZF, Nur77, and SOX4), the epigenetic regulator and histone demethylase UTX, autophagy-related gene 7 (Atg7), and the microRNA miR-181^31–38^. Thus, a precise and unique machinery that differs from that used by conventional T cells is required for *i*NKT-cell development.

After thymic development, *i*NKT cells acquire some memory characteristics and exit the thymus^39^. They then travel to the peripheral tissues, where the *i*NKT pool is maintained unless cued otherwise^26,40^. Notably, the thymic *i*NKT-cell subsets (i.e., *i*NKT1, *i*NKT2, and *i*NKT17 cells) display different patterns of peripheral localization. For example, *i*NKT1 cells account for most of the *i*NKT cells in the liver, with the other subsets being infrequent. This may be due to their expression of different chemokine receptors and integrins^41^.

## General characteristics of pulmonary *i*NKT cells

In the mouse lung, *i*NKT cells account for ~5% of the resident lymphocytes and localize in the interstitial space as well as the vasculature of the lungs^42^. All three *i*NKT-cell subsets are present in the lung. Interestingly, RNA sequencing analysis of *i*NKT cells in various tissues showed that pulmonary *i*NKT subsets share common characteristics in terms of their transcriptome profiles that distinguish them from *i*NKT cells in other tissues^6,43^. Specifically, all pulmonary *i*NKT cells, but not other *i*NKT cells, display high expression of AP-1, other bZIP family members, some NF-κB family members, CTLA-4, CD69, and Nur77^43^. It is likely that this unique transcriptome profile is driven by the homeostatic lung microenvironment since Lee et al. showed that the profile was unchanged by local infection or inflammation^6,43^. This notion is further supported by the fact that lung-resident mucosa-associated invariant T (MAIT) cells, γδ T cells, and alveolar macrophages also bear lung-specific signatures that are different from those of the corresponding cells in other tissues^44–46^.

## Localization of pulmonary *i*NKT cells

**Fig. 1:**
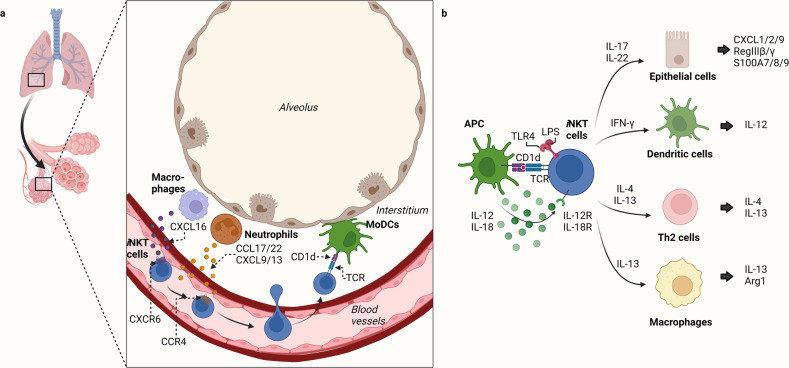
Distribution of pulmonary *i*NKT cells and their interactions with other cells in the lung. **a** Migration of *i*NKT cells to the lungs. After developing in the thymus, *i*NKT cells express CXCR6, a tissue localization molecule, and migrate to the CXCL16-expressing periphery. iNKT cells accumulate in the lumen of the lung microvasculature and then enter the lung tissue when neutrophils in the lung interstitium secrete the CCR4 ligands CCL17/22 and CXCL9/13. The neutrophils, therefore, guide the iNKT cells to the source of lung injury in the interstitium. The monocyte-derived DCs (moDCs) in this area present glycolipid antigens from the lung injury source to the *i*NKT cells, which become activated and then remain in the interstitium. **b** Crosstalk between *i*NKT cells and other cells in the lung. *i*NKT cells are activated by antigens expressed by lung antigen-presenting cells (APCs), such as MoDCs, by cytokines from other cells (such as IL-12/18), and by TLR ligands (e.g., LPS). The activated *i*NKT cells then secrete a variety of cytokines that regulate the function of many types of neighboring cells.

After moving from the thymus into the circulation, *i*NKT cells accumulate in the lung microvasculature. When the lung tissue is stimulated by airborne antigens or infections that bear the glycolipid or microbial membrane component targets of *i*NKT cells, the *i*NKT cells extravasate into the interstitium and bronchiolo-alveolar spaces^42^. This is not observed in other organs; for example, the large numbers of *i*NKT cells in the liver remain within the microvasculature^40^. The trafficking of *i*NKT cells to the lung and their extravasation into the lung appears to be driven by (i) the chemokines that are generated by the lung tissue when it encounters airborne *i*NKT-cell ligands^42^ and (ii) *i*NKT-cell expression of a specific chemokine-receptor profile^47^. The chemokines include thymus-and-activation-regulated chemokine (TARC, also known as CCL17), MIG/CXCL9, and BCA-1/CXCL13^41,48,49^, while the *i*NKT-cell chemokine receptors include CCR4, CCR9, and CXCR6^47^ (Fig. 1a). CCR4, in particular, appears to play a crucial role in *i*NKT-cell migration to the lungs and airways and the subsequent redistribution of *i*NKT cells within the lung. For example, aerosol administration of exogenous CCR4 ligand (TARC/CCL17) causes *i*NKT cells to promptly extravasate into the lung tissue^42^. Moreover, antibody-mediated neutralization of CCR4 or CCR4 deletion blocks *i*NKT cell migration to the lungs, thereby attenuating the airway hyperresponsiveness (AHR) induced in mice by pulmonary administration of antigen or αGalCer^47–49^. In addition, asthma patients have elevated levels of both CCR4 ligands (TARC/CCL17 and CCL22, which is also known as macrophage-derived chemokine) in their bronchoalveolar lavage fluid (BALF)^50–53^. Similarly, mice that are deficient in one component of another key pulmonary *i*NKT-cell chemokine/chemokine-receptor combination, namely, CXCR6 and its ligand CXCL16, bear significantly fewer *i*NKT cells within the intravascular compartment of the lungs^40,42^. Similar observations have been made for CCR9, the third pulmonary *i*NKT-cell chemokine receptor^54–56^.

Notably, intravital imaging of the lung tissue showed that neutrophils play a key role in the α-GalCer- or *Streptococcus pneumoniae* infection-induced migration of pulmonary *i*NKT cells from the vasculature into the interstitium; specifically, neutrophils produce CCL17, which guides the migration of *i*NKT cells through the lung tissue. Antigen-presenting cells (APCs), including monocyte-derived DCs, also play an important role in *i*NKT-cell migration: they promote neutrophil extravasation into the lung and present antigen to *i*NKT cells, thereby halting further migration of these cells^57^ (Fig. 1a).

## Activation of pulmonary *i*NKT cells

*i*NKT cells are most often activated by recognizing glycolipid antigens presented on CD1d^58^ (Fig. 1b). While many immune cells express CD1d on their surface, DCs are the most potent APCs in terms of glycolipid:CD1d-induced *i*NKT-cell activation^59,60^. While the most potent glycolipid identified to date is α-GalCer^61^, several studies have shown that *i*NKT cells can also recognize glycolipids from various microorganisms^62^. For example, the transfer of *Sphingomonas*-pulsed DCs into mice successfully activated lung-resident *i*NKT cells; the stimulatory ligands were found to be α-galacturonosylceramide and α-glucuronosylceramide^63^. Moreover, mycobacterium-derived phosphatidylinositol mannoside can activate *i*NKT cells^64^. Thus, despite the restricted TCR repertoire of *i*NKT cells, they may recognize a wider range of glycolipid antigens than initially thought.

Another common mechanism by which *i*NKT cells are activated is the signaling induced by cytokines^65^ from surrounding immune cells, particularly DCs^66^ (Fig. 1b). For example, when DCs are activated by the engagement of Toll-like receptors (TLRs) on their surface, they produce IL-12, which successfully activates pulmonary *i*NKT cells even when CD1d is absent^67^. The importance of this mechanism is supported by the constitutive expression of the IL-12 receptor of *i*NKT cells and the fact that they rapidly upregulate their STAT4 expression when they encounter IL-12. Similarly, IL-18 and type-1 interferon produced by DCs can also induce pulmonary *i*NKT cells to secrete their own cytokines^68–70^.

Finally, *i*NKT cells can be activated by their own innate immune sensors, including TLR4. Flow cytometry and confocal microscopy analyses show that *i*NKT cells express TLR4 on their surface and in their endosomes and that simultaneously stimulating TCR and TLR4 on *i*NKT cells increases their expression of IFN-γ. Notably, this dual stimulation also decreases *i*NKT-cell expression of IL-4, which suggests that the TLR4 signaling pathway skews *i*NKT cells toward type-1 immune responses. Notably, this regulatory skewing appears to be particularly important for several lung diseases. For example, we showed that while the adoptive transfer of LPS-treated *i*NKT cells (LPS stimulates TLR4) suppresses pulmonary fibrosis, it worsens hypersensitivity pneumonitis^71^.

Taken together, these studies suggest that pulmonary *i*NKT cells can be activated via multiple mechanisms, although the mechanism that plays the most prominent role in lung health and disease remains to be determined. These observations also suggest that pulmonary *i*NKT cells may be readily activated in pathological environments that bear *i*NKT-cell antigens, cytokines from other immune cells, and/or TLR-activating molecules. Indeed, it is likely that such environments activate pulmonary *i*NKT cells via several or all of these mechanisms. Further improving our understanding of these mechanisms is important for enhancing our understanding of pulmonary *i*NKT cells.

## Functions of pulmonary *i*NKT cells

*i*NKT cells exert their immune effects in vivo via several mechanisms. The most prominent involves *i*NKT-cell secretion of cytokines and other soluble factors^65^. Consequently, most studies on *i*NKT-cell functions have focused on this mechanism. Given that multiple *i*NKT-cell subsets exist (i.e., *i*NKT1, *i*NKT2, *i*NKT17, and *i*NKT10), *i*NKT cells are considered multipotent cytokine-secreting cells. This ability to secrete powerful cytokines with opposing properties also suggests that these cells can regulate the cytokine production of neighboring immune cells in various ways, thus controlling the entire cytokine milieu (Fig. 1b). Indeed, there are many cases of this in the literature. For example, *i*NKT cells secrete granulocyte-macrophage colony-stimulating factor (GM-CSF), which promotes macrophage production of IL-1β. Conversely, they also express IL-4, which promotes M2-macrophage differentiation; this inhibits macrophage secretion of IL-1β in vitro^11^. Another example is that the IL-4 secreted by *i*NKT cells can suppress the IFN-γ production of neutrophils in the respiratory tract^72^. Conversely, *i*NKT cells can themselves secrete IFN-γ, which in turn regulates neutrophil production of IL-10 and C5a in a sepsis model^73^. Thus, *i*NKT cells have the clear potential to balance immune responses via their versatile secretion of powerful cytokines.

Indeed, the cytokine production of *i*NKT cells has been shown to facilitate or even play a critical role in immune diseases such as autoimmunity, allergy, infection, and cancer^6,65,74–76^. More specifically, the cytokine production of *i*NKT cells has been implicated in numerous lung conditions, including allergic asthma^77^, mycobacterium infection^78^, viral infection^79^, chronic obstructive pulmonary disease (COPD)^80^, pulmonary fibrosis^81^, hypersensitivity pneumonitis^72^, and immune complex-induced lung injury^82^. Of the many soluble factors that pulmonary *i*NKT cells can secrete, IL-4 and IFN-γ appear to play particularly important roles in respiratory tract diseases^83,84^. Other important soluble factors include IL-17, IL-22, and IL-13, which regulate the activities of neighboring cells such as helper T cells^85^, lung epithelial cells^86^, DCs^13,87^, and macrophages^88^. The roles of these factors in lung disease are detailed further below (Fig. 1b).

Other mechanisms by which *i*NKT cells exert immune effects include cytotoxicity and cell-to-cell contact-mediated immune regulation^89^. However, research on the roles of these mechanisms in lung diseases is lacking.

It should be noted here that *i*NKT cells can play both beneficial roles, such as protecting the lungs from tuberculosis (TB)^90^, and detrimental roles, such as driving COPD by producing IL-17^91^. Further research on the roles of pulmonary *i*NKT cells in respiratory tract diseases is likely to be particularly valuable because it may promote the development of novel therapeutic targets.

## Metabolism of pulmonary *i*NKT cells

Although little is known about the metabolism of pulmonary *i*NKT cells, it can be inferred by examining the metabolic properties of peripheral *i*NKT cells and the metabolic environment of the lung. Before we discuss these points, we will first describe what is known about the metabolism of conventional T cells. These cells first engage in glycolytic metabolism, which is induced by PI3K-Akt signals from the pre-TCR and Notch1 during β-selection in the thymus^92^. Thereafter, mature naïve quiescent T cells in the periphery primarily utilize oxidative phosphorylation (OXPHOS) or fatty acid oxidation (FAO) to generate ATP; thus, fatty acids are their main energy source. However, once T cells are activated, they switch to aerobic glycolysis^93^ for ATP production and primarily use glucose as their fuel source. While this is less efficient than glycolysis, this switch provides the rapid energy needed for effector T-cell long-term survival, proliferation, cytokine secretion, and migration to sites of inflammation^92,94^.

Although the proliferation of developing *i*NKT cells appears to rely on glucose utilization, glycolysis has been suggested to exert a negative effect on the function of *i*NKT cells since glucose uptake and Glut1 expression are higher in immature *i*NKT cells present in the thymus than in mature *i*NKT cells^34,95^. Glucose uptake and Glut1 expression are attenuated in mature *i*NKT cells and upregulated when there are defects in developmental factors of *i*NKT cells, including Keap1 or PLZF^34,96,97^. Furthermore, *i*NKT cells are more susceptible to modifications in the function of the mitochondrial electron transport chain than conventional T cells. The crucial role of mitochondrial metabolism in the development and function of *i*NKT cells is exerted through the regulation of TCR/IL-15 signaling and NFAT activity^98^.

Peripheral *i*NKT cells bear memory T-cell features such as high cell surface expression of CD44 and retention of antigen specificity after maturation and antigen exposure^99^. Moreover, these cells are in a ready-to-respond state that allows them to produce cytokines within hours, even when they are only stimulated by interleukins such as IL-12 and IL-18^100^. Thus, peripheral *i*NKT cells are similar to memory T cells in that their metabolism allows rapid responsiveness. However, while conventional effector/memory, CD4^+^ T cells use glucose for glycolysis, which leads to lactate production, *i*NKT cells metabolize glucose via the pentose phosphate pathway and OXPHOS, and these pathways are essential for their survival, proliferation, and cytokine production^96^. This is supported by the fact that *i*NKT cells have higher ATP levels than CD4^+^ T cells both before and after activation^96^. Moreover, peripheral *i*NKT cells display lower glucose uptake than conventional CD4^+^ T cells due to the inhibitory effect of PLZF on glycolysis^96^. Finally, although both CD4^+^ T and *i*NKT cells require glutamine to proliferate, CD4^+^ T cells need glucose to expand optimally, whereas *i*NKT cells depend on fatty acid metabolism^96^.

The metabolism of *i*NKT cells in the lungs may be shaped by the metabolic environment in the lung. This environment is determined by several dynamic and complex factors, including mucus and microbacterial components. The mucus is produced by the airway epithelium. Since it is a rich source of nutrients for bacteria, it can affect the metabolic activity of these microorganisms^101^. Moreover, microbacterial components such as LPS and other bacterial byproducts can create a complex metabolic environment that alters the local pH, oxygen levels, and nutrient availability. This can affect the metabolism and consequent energy production and cellular behavior of not only the microorganisms but also the host cells^102^, including local *i*NKT cells. It seems likely that pulmonary *i*NKT cells are characterized by a specific metabolomic profile that promotes their use of metabolic pathways such as fatty acid metabolism.

*i*NKT-cell responses are now known to be highly dependent on their synthesis of lipids. For example, compared to conventional T cells, *i*NKT cells express higher levels of PPAR-γ, a master regulator of lipid metabolism. They also increase their cholesterol synthesis after activation, which is needed for their TCR signaling, proliferation, and production of IFN-γ. Interestingly, however, blocking cholesterol synthesis only slightly diminished the production of IL-4 by activated *i*NKT cells; rather, glucose appeared to be more critical for IL-4 production by *i*NKT cells^103^. The influence of lipid biosynthesis on *i*NKT-cell function is highlighted by the fact that *i*NKT cells skew toward an *i*NKT1 phenotype.

## *i*NKT cells in pulmonary diseases

Despite their relatively low numbers in the lungs, pulmonary *i*NKT cells appear to play vital roles in host defense against microorganisms. This role involves them patrolling the lumen of the pulmonary vessels and the interstitial tissue until the lung tissue is injured by infection and emits danger signals. Such signals cause *i*NKT cells to migrate to the injured site, which contains glycolipid antigens, and induce *i*NKT cells to elicit early host defense mechanisms. However, pulmonary *i*NKT cells can also participate in the pathogenesis of various lung diseases^104^, either via direct pathogenic effects of their cytokines or more indirectly via regulation of neighboring immune-cell subset functions^105^. The triggers that generate pathogenic *i*NKT-cell activity are generally the same as those that initiate protective *i*NKT-cell responses, namely, glycolipid antigens and/or the surrounding cytokine milieu. Below, we will summarize what is known about the role of *i*NKT cells in the four most common lung diseases (asthma, mycobacterium infection, viral infection, and COPD) as well as several more minor pulmonary diseases.

### Asthma

Asthma is a common respiratory disease that affects millions of people worldwide^104^. Its cardinal feature is AHR, but there are several distinct forms of asthma that are underpinned by disparate pathogenic pathways^106,107^. The most common form is allergic asthma, which is triggered by allergens and is characterized by Th2-immune responses, eosinophil infiltration, high IgE levels, and AHR^108^. Another important endotype is nonallergic asthma, which is Th2-independent and characterized by lung/airway neutrophil infiltration, Th17-immune responses, and AHR^109^.

**Fig. 2:**
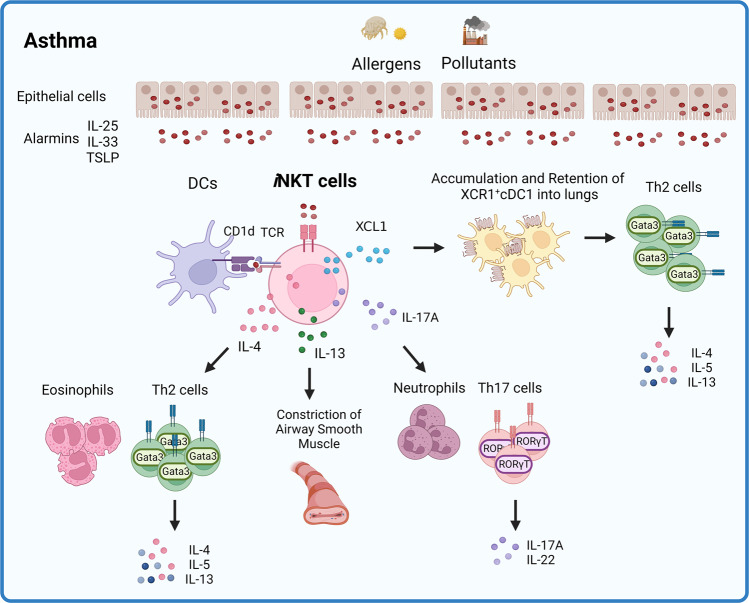
Roles of pulmonary *i*NKT cells in asthma pathogenesis. Inhaled allergens or pollutants cause lung epithelial cells to release alarmins and dendritic cells (DCs) to present the antigens contained in these environmental insults. Both the alarmins and the DCs then activate *i*NKT cells. Activated *i*NKT cells subsequently secrete cytokines that modulate the function of various immune cells or cause airway smooth muscle constriction. In particular, *i*NKT cells promote the recruitment and lung retention of XCR1-expressing cDC1s by secreting the chemokine XCL1. The XCR1^+^ cDC1s subsequently amplify the type-2 immune responses during asthma.

Studies in mice^110–112^ and nonhuman primates^113^ show that *i*NKT cells can directly trigger the development of asthma. For example, intranasal administration of α-GalCer^114^ or *Sphingomonas*-derived glycolipids in mice induces AHR^54^. Moreover, IL-4 and particularly IL-13 from *i*NKT cells are key drivers of allergic asthma^83^: IL-4 facilitates the overall Th2 response in allergic lungs, while IL-13 acts as a direct pathogenic factor^83^ by inducing lung epithelial cell contraction^115^. The triggers for IL-4/IL-10 secretion by *i*NKT cells are IL-25, thymic stromal lymphopoietin (TSLP), and IL-33: these so-called ‘alarmin’ cytokines are released by injured lung cells, can directly activate *i*NKT cells and are abundant in early asthmatic lungs^116^. Similarly, *i*NKT cells help drive the development of Th17-mediated asthma. While less is known about the role of *i*NKT cells^117,118^, it has been shown that intranasal administration of α-GalCer causes pulmonary CD4^−^NK1.1^−^ *i*NKT cells to secrete IL-17^119^, which promotes airway neutrophilia and AHR. This was also observed when a more physiological model of IL-25-dependent AHR was employed^120^ (Fig. 2).

Our recent study^13^ on the role of *i*NKT cells in murine allergic asthma models induced by ovalbumin (OVA) and house-dust mite (HDM) suggests that *i*NKT cells also promote the development of allergic asthma by enhancing the migration of XCR1-expressing type-1 conventional DCs (cDC1s) into asthmatic lungs. This migration appears to be driven by *i*NKT-cell production of X-C-motif chemokine ligand 1 (XCL1). This ligand may be specific for cDC1s since they are the only immune-cell subset that appears to express the receptor for XCL1 (XCR1). The importance of this *i*NKT cell-cDC1 relationship was demonstrated by the fact that XCL1-knockout (KO), XCR1-KO, and Jα18-KO (which lack *i*NKT cells) mice failed to develop AHR and Th2-immune responses, which was associated with diminished cDC1 infiltration into the lungs. This *i*NKT cell-cDC1 relationship was confirmed by adoptively transferring wild-type (WT) or XCL1-deficient *i*NKT cells into Jα18-KO mice: the WT cells, but not the XCL1-deficient cells, induced AHR and cDC1 migration into the lungs. Similarly, adoptive transfer of WT cDC1s into XCL1-KO, XCR1-KO, or Jα18-KO mice induced AHR. Notably, we also showed that once cDC1s entered the lungs, they activated the Th2 responses of CD4^+^ T cells^13^. This is interesting because cDC1s are generally thought to regulate CD8^+^ T-cell responses^121^. Our finding is supported by Nakano et al., who also showed that cDC1s can regulate the Th2 responses of CD4^+^ T cells in allergic asthma^122^. Thus, *i*NKT cells play critical roles in the murine asthma model by not only generating directly pathogenic cytokines but also recruiting key cells that then evoke the pathogenic activities of Th2 T cells (Fig. 2).

It should be noted that there is some controversy about the importance of *i*NKT cells in murine asthma models^123^ because several studies have shown that KO of Jα18 or CD1d (and therefore *i*NKT cells) has no effect on the development of asthma in commonly used murine models (the OVA and HDM models)^124,125^. This may reflect differences in microbiota between animal facilities since germ-free mice show increased pulmonary *i*NKT-cell numbers^126^. However, the role of *i*NKT cells in asthma is even more controversial in humans. While Akbari et al. showed that the BALF of patients with moderate to severe asthma had very high frequencies of *i*NKT cells (60% of CD3^+^ cells)^83^, other groups observed frequencies of only ~2%. These disparities may reflect improper flow cytometric analysis, nonspecific binding of the CD1d tetramers used, or differences in patient cohorts. As an aside, the BALF of childhood asthma patients bears increased *i*NKT-cell numbers^127^, which suggests that *i*NKT cells may participate in juvenile asthma. Overall, it seems possible that *i*NKT cells promote asthma, although this role may only emerge in specific conditions.

### Mycobacterium infection

TB remains a serious public health concern worldwide: approximately 2 million people die per year from this disease^128^. This reflects the high contagiousness of the pathogenic *Mycobacterium* species (*M. tuberculosis*, *M. bovis*, *M. microti*, and *M. africanum*^129^) and the poor protection generated by the Bacillus Calmette–Guerin vaccine in adults^130^.

**Fig. 3:**
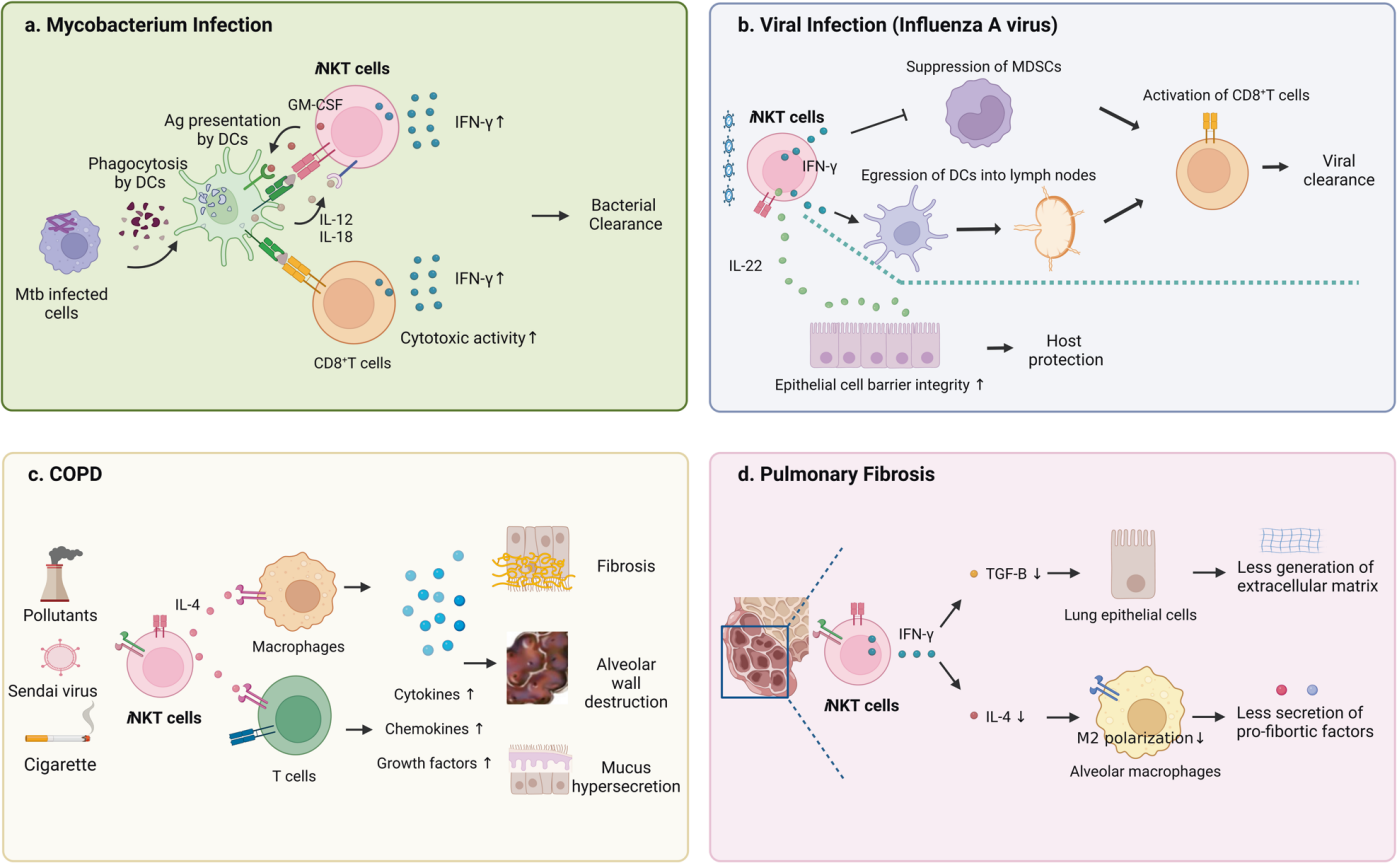
Role of pulmonary *i*NKT cells in various lung diseases. **a** In mycobacterium infection, cDC1s activate pulmonary *i*NKT cells by secreting IL-12 and IL-18 and by presenting antigen on CD1d. The *i*NKT cells then reciprocally stimulate the cDC1s via GM-CSF, which activates the cytotoxic CD8^+^ T cells that eliminate the infected cells and clear the bacteria. This activity is further supported by the IFN-γ produced by the iNKT cells. **b** During lung viral infections, pulmonary *i*NKT cells produce IFN-γ, which downregulates myeloid-derived suppressor cells (MDSCs), which would otherwise suppress CD8^+^ T cells. Moreover, *i*NKT cells promote the maturation of DCs and their migration to draining lymph nodes by IFN-γ, which enhances CD8^+^ T-cell responses. Finally, *i*NKT cells protect the host by increasing epithelial cell integrity by producing IL-22. **c** COPD pathogenesis is promoted by IL-4-secreting *i*NKT cells. IL-4 generates IL-13-secreting macrophages and activates T cells, which in turn secrete many cytokines, chemokines, and growth factors that directly induce fibrosis, alveolar wall destruction, and mucus hypersecretion. **d** Pulmonary fibrosis is inhibited by *i*NKT cells. The mechanism involves *i*NKT-cell expression of IFN-γ, which suppresses the production of the master profibrotic regulator TGF-β in the lung. This blocks the extracellular matrix (ECM) production that is responsible for pulmonary fibrosis. Another mechanism involves downregulating lung expression of IL-4, which inhibits the polarization of lung macrophages to the M2 phenotype. This phenotype plays an important role in fibrosis by producing type-2 cytokines and large amounts of TGF-β.

The role of *i*NKT cells in TB has been studied in murine models. *i*NKT cells in infected mice are activated by not only CD1d-presented mycobacterial antigen^131^ but also cytokines in the environment such as IL-12 and IL-18^132^: the resulting activated *i*NKT cells help protect the mice from the infection^78^. This role is partly mediated by the GM-CSF secretion of *i*NKT cells: this activates cDC1s, which in turn promote the antimicrobial activities of CD8^+^ T cells^133^. Another mechanism is that *i*NKT cells facilitate the priming of T cells against TB infection: treatment of TB-infected mice with α-GalCer increases the numbers of TB antigen-specific IFN-γ-producing T cells in the lung^134^. Finally, it is possible that *i*NKT cells exert their protective abilities in TB by releasing IFN-γ and engaging in cytotoxicity: when *i*NKT cells from *M. tuberculosis* or *M. bovis-*infected mice were cocultured in vitro with infected macrophages, the *i*NKT cells secreted IFN-γ and killed the macrophages^132^ (Fig. 3a). However, this function remains to be validated in vivo.

With regard to humans, the *i*NKT cells in the sputum of TB patients bear activated phenotypes and secrete high IFN-γ levels^135^. However, their numbers are significantly decreased. Interestingly, these cells express high levels of PD-1 on their surface and have a tendency to undergo apoptosis. Indeed, when these cells were treated in vitro with a PD-1 blocker, the *i*NKT-cell frequency rose^136^. Moreover, the decreased *i*NKT-cell numbers in the blood of humans normalize after TB is eliminated by treatment^137^. Notably, cocultured human *i*NKT cells can also kill *M. tuberculosis-*infected macrophages in vitro^138^. This supports the murine in vitro study above that suggests *i*NKT cells can protect the host from TB infection via cytotoxicity^132^.

These preclinical and clinical studies suggest that *i*NKT cells can protect against TB infection. Thus, cell-based therapies with *i*NKT cells may be useful for TB patients^139^. This is supported by a study showing that when *i*NKT cells were activated exogenously with α-GalCer and then transferred into mice, the mice were protected from lethal TB infection^140^. Another possibility is to incorporate α-GalCer into the Bacillus Calmette–Guerin vaccine: this strategy has been shown to enhance the overall immune response of TB-infected mice^134^.

### Viral infections

#### Influenza A virus

Influenza A virus (IAV) is another common cause of respiratory tract disease. It is especially prominent during winter and can cause severe and sometimes fatal lung damage. It can also increase host susceptibility to secondary bacterial infections^141^.

Pulmonary *i*NKT cells protect mice from the H1N1 and H3N2 IAV strains since *i*NKT cell deficiency (Jα18-KO and CD1d-KO) resulted in more severe bronchopneumonia, greater weight loss, and shorter time to death^142,143^. Moreover, treatment with exogenous α-GalCer before IAV infection reduces infection-induced weight loss and increases CD8^+^ T- and NK-cell responses to IAV infection; the latter effects are due to IFN-γ secreted by *i*NKT cells^144^ (Fig. 3b). There are also several other mechanisms by which *i*NKT cells protect the host from IAV. First, they downregulate myeloid-derived suppressor cells, which inhibit the CD8^+^ T-cell responses that limit IAV replication during moderate H1N1 infection^143^. Second, they promote the maturation of DCs and their migration to draining lymph nodes, which enhances CD8^+^ T-cell responses during severe H3N2 infection^142^. Third, *i*NKT cells protect the host from excessive lung damage by producing IL-22, which may strengthen the epithelial barrier integrity of host lungs^86^. Fourth, *i*NKT cells reduce the local levels of MCP-1. This decrease downregulates the accumulation of inflammatory monocytes in the lungs, which diminishes the overall damage to the lung tissue. This, in turn, promotes the survival of mice infected with a highly pathogenic strain of IAV^88^. Thus, *i*NKT cells help block the deleterious effects of IAV infection by both promoting anti-viral responses and protecting the lung tissue of the host (Fig. 3b).

#### COVID-19

Only a few studies have assessed the role of *i*NKT cells in the recent pandemic caused by SARS-CoV-2 infection. One recent study reported that patients with severe COVID-19, but not patients with mild COVID-19, demonstrate a tenfold reduction in pulmonary *i*NKT-cell frequencies^145^. However, another group found that COVID-19 patients and healthy individuals did not differ markedly in *i*NKT-cell frequencies^146^. Another study showed that COVID-19-mediated pneumonia was associated with elevated eosinophil and neutrophil numbers in the sputum and BALF and that these numbers correlated positively with the numbers of *i*NKT cells in peripheral blood and BALF. This suggests that *i*NKT cells could play a pathogenic role in COVID-19^147^. Finally, hematopoietic stem cell (HSC)-engineered *i*NKT cells, which were differentiated from TCR-engineered HSCs, efficiently killed SARS-CoV-2-infected cells in vitro^148^. Thus, *i*NKT cells may help protect the host from SARS-CoV-2 infection by killing infected cells. These observations together suggest that *i*NKT cells are involved in COVID-19-related immune responses. However, whether their role is primarily protective or pathogenic remains to be determined.

#### Chronic obstructive pulmonary disorder (COPD)

The frequency and severity of COPD has steadily increased over the last few decades, and COPD now affects more than 200 million people worldwide and is the fourth leading cause of death^149^. COPD is characterized by lung tissue emphysema, respiratory bronchiolitis, and eventual chronic bronchitis^150^.

Key risk factors for COPD are cigarette smoke and industrial pollutants^150^. Several studies suggest that *i*NKT cells play pathogenic roles in COPD. First, the murine cigarette smoke model of COPD features increased pulmonary *i*NKT-cell numbers. Second, both Jα18-KO and CD1d-KO mice are resistant to forming COPD based on exposure to cigarette smoke^80^. Third, repeated intranasal administration of α-GalCer can induce COPD, and this is ameliorated when an anti-IL-4 antibody is coadministered^80^. Fourth, consistent with the mouse COPD model, COPD patients have greater *i*NKT-cell numbers in their peripheral blood and sputum than healthy individuals^151^. Fifth, *i*NKT cells from COPD patients produce high levels of IFN-γ and IL-17A that can activate cigarette smoke extract-exposed lung DCs or airway epithelial cells in vitro^91^. Sixth, *i*NKT-cell deficiency ameliorates symptoms in the Sendai virus infection model of COPD. This is mediated by *i*NKT-cell downregulation of the numbers of IL-13^+^ macrophages, which are critical COPD regulators because IL-13 derived from these cells directly induces goblet cell metaplasia, AHR, and mucus production. This mechanism may also participate in human COPD since the lungs of COPD patients demonstrate elevated numbers of not only *i*NKT cells but also IL-13^+^ CD68^+^ macrophages^152^ (Fig. 3c). Thus, it is likely that *i*NKT cells promote COPD pathogenesis, possibly by direct and/or indirect interactions with IL-13^+^ macrophages.

### Pulmonary fibrosis, hypersensitivity pneumonia, and immune complex-induced lung injury

Our studies suggest that pulmonary *i*NKT cells also play critical protective or pathogenic roles in pulmonary fibrosis, hypersensitivity pneumonitis, and immune complex-induced lung injury, as detailed below.

#### Pulmonary fibrosis

Pulmonary fibrosis is characterized by progressive scarring of the lungs and the eventual development of lung interstitial fibrosis. This results in progressive shortness of breath^153^. Since this disease is caused by excessive extracellular matrix (ECM) production by fibroblasts in the lungs^154^, the recruitment, proliferation, and ECM production of fibroblasts have been extensively studied. At present, it is thought that these fibroblast activities are largely driven by transforming growth factor beta (TGF-β), which is a potent profibrotic mediator^155^.

Two studies suggest that *i*NKT cells can play protective roles in pulmonary fibrosis by downregulating TGF-β expression^81,156^. First, we found that CD1d-KO mice developed more severe bleomycin-induced pulmonary fibrosis than WT mice, as shown by their worse lung histology, higher hydroxyproline levels, and greater mortality^64^. These severe effects were associated with higher TGF-β levels and lower IFN-γ levels in the lungs. Moreover, the adoptive transfer of WT *i*NKT cells into CD1d-KO mice not only ameliorated the severity of the disease in these mice but also restored the IFN-γ levels in the lung while concomitantly reducing the TGF-β levels. Interestingly, when bleomycin-treated BALF cells were treated in vitro with recombinant IFN-γ, their production of TGF-β dropped markedly. These findings suggest that IFN-γ-secreting *i*NKT cells help protect against pulmonary fibrosis by downregulating TGF-β1 expression in the fibrotic lung^81^. Our findings are supported by Grabarz et al., who observed that when WT mice were injected intratracheally with bleomycin and intraperitoneally with α-GalCer on the same day, pulmonary fibrosis was less severe. This was associated with lower IL-4 levels in the lung and lower expression of arginase-1 by neighboring alveolar macrophages. These findings suggest that *i*NKT cells may protect mice from pulmonary fibrosis by suppressing lung production of IL-4, which inhibits M2-macrophage polarization^156^. This is significant because the recruitment of monocytes to the lung and their conversion into type-2 cytokine- and TGF-β-secreting M2 macrophages drive the overall progression of pulmonary fibrosis^157^. Thus, *i*NKT cells protect mice from pulmonary fibrosis by (i) producing IFN-γ, thereby downregulating lung TGF-β levels and (ii) decreasing lung IL-4 levels, thereby inhibiting M2-macrophage activity (Fig. 3d).

#### Hypersensitivity pneumonitis

Hypersensitivity pneumonitis is caused by repetitive exposure to inhaled environmental antigens that provoke a hyperreactive immune response. This response induces inflammation of the alveoli and bronchioles and often leads to other interstitial lung diseases^158^. There is little in the literature about the roles of *i*NKT cells in hypersensitivity pneumonitis. However, we found that CD1d-KO mice were more susceptible to *Saccharopolyspora rectivirgula*-induced hypersensitivity pneumonitis than WT mice and that this was associated with elevated IFN-γ levels in the lung. IFN-γ was mainly produced by Gr-1^+^ neutrophils and played an important pathogenic role since blocking IFN-γ or depleting Gr-1^+^ neutrophils attenuated hypersensitivity pneumonitis-associated inflammation in CD1d-KO mice. Additional experiments then showed that the production of IFN-γ by Gr-1^+^ neutrophils in hypersensitivity pneumonitis was impaired by IL-4 produced by *i*NKT cells: adoptive transfer of IFN-γ-deficient, but not IL-4-deficient, *i*NKT cells downregulated hypersensitivity pneumonitis-related inflammation in CD1d-KO mice^72^.

#### Immune complex-induced lung injury

IgM or IgG immune complexes (ICs) are critical regulators of the immune system. However, they can cause acute respiratory distress syndrome (ARDS) or acute lung injury (ALI) due to unwanted inflammation^159^. We showed that CD1d-KO and Jα18-KO mice are less susceptible to developing ALI in the chicken egg albumin- and anti-chicken egg albumin IgG-induced IC-ALI model than WT mice. Additional experiments showed that the IC-ALI in WT mice was induced by *i*NKT cells, whose secretion of IFN-γ and macrophage inflammatory protein-1α (MIP-1α) caused neighboring alveolar macrophages and DCs to secrete proinflammatory cytokines. In particular, adoptive transfer of WT *i*NKT cells into CD1d-KO mice generated the IC-ALI seen in WT mice, but this was not observed when IFN-γ-deficient, MIP-1α-deficient, or FcγR-deficient *i*NKT cells were transferred^82^.

Altogether, these studies show that along with their crucial roles in common respiratory tract diseases, pulmonary *i*NKT cells are also important in many other respiratory tract diseases. These findings demonstrate the importance of these cells in governing overall lung immunity.

## Concluding remarks

This review sought to provide a broad perspective on pulmonary *i*NKT cells and their roles in lung diseases that will hopefully aid further research on these cells. While it is clear that these cells are important for protection from lung infections and play important roles in the pathogenesis of many lung diseases, there are still many missing links. It is increasingly evident that overall immunity is driven by very complex and dynamic interactions between a wide variety of immune cells. Given the cytokine multipotency of *i*NKT cells and their multiple functions, it is likely that further research on the interrelationships between pulmonary *i*NKT cells and surrounding immune cells will be fruitful in terms of improving our understanding of the immune mechanisms that protect and harm the lungs.
